# Protection against Foot-and-Mouth Disease Virus in Guinea Pigs via Oral Administration of Recombinant *Lactobacillus plantarum* Expressing VP1

**DOI:** 10.1371/journal.pone.0143750

**Published:** 2015-12-02

**Authors:** Miao Wang, Li Pan, Peng Zhou, Jianliang Lv, Zhongwang Zhang, Yonglu Wang, Yongguang Zhang

**Affiliations:** 1 State Key Laboratory of Veterinary Etiological Biology, National Foot-and-Mouth Disease Reference Laboratory, Lanzhou Veterinary Research Institute, Chinese Academy of Agricultural Sciences, Lanzhou, Gansu, China; 2 Jiangsu Co-innovation Center for Prevention and Control of Important Animal Infectious Diseases and Zoonoses, Yangzhou, Jiangsu, China; Institut National de la Santé et de la Recherche Médicale (INSERM), FRANCE

## Abstract

Mucosal vaccination is an effective strategy for generating antigen-specific immune responses against mucosal infections of foot-and-mouth disease virus (FMDV). In this study, *Lactobacillus plantarum* strains NC8 and WCFS1 were used as oral delivery vehicles containing a pSIP411-VP1 recombinant plasmid to initiate mucosal and systemic immune responses in guinea pigs. Guinea pigs were orally vaccinated (three doses) with NC8-pSIP411, NC8-pSIP411-VP1, WCFS1-pSIP411, WCFS1-pSIP411-VP1 or milk. Animals immunized with NC8-pSIP411-VP1 and WCFS1-pSIP411-VP1 developed high levels of antigen-specific serum IgG, IgA, IgM, mucosal secretory IgA (sIgA) and neutralizing antibodies, and revealed stronger cell-mediated immune responses and enhanced protection against FMDV challenge compared with control groups. The recombinant pSIP411-VP1 effectively improved immunoprotection against FMDV in guinea pigs.

## Introduction

Foot-and-mouth disease (FMD) is a highly contagious disease of livestock. Outbreaks of FMD cause severe financial losses [1], and often lead to quarantining and export limitations in affected countries, as well as culling of herds. After a 2–12 day incubation period, affected animals develop acute onset of high fever, which is followed by vesicle formation in the mouth, pharynx and on the feet. Affected animals suffer pain, refuse their feed, and salivate extensively. The causal pathogen, FMDV, belongs to the genus *Aphthovirus* of the family *Picornaviridae* and includes seven serotypes, A, O, C, Asia 1, SAT1, SAT2 and SAT3. The virion has a high potential for genetic and antigenic variation. Cross-protective antibodies are not formed after infection or vaccination by other serotypes and subtypes of FMDV. This has confounded the efforts of vaccination programs for preventing the disease [2]. The viral genome is a positive single-stranded RNA, with a protein coat consisting of four capsid proteins enumerated as VP1, VP2, VP3, and VP4. Currently available parenteral vaccines employed to control FMD are inactivated and contain the whole virus in a semi-purified state, especially the VP1 structural polypeptide, forming the virion as the immunological component, which shows the promising protection for animals against FMD [3].

Mucosal vaccines have been found to induce sufficient mucosal responses to prevent the virus from establishing the mucosa as a site of continued replication and dissemination [4]. Mucosal immunization can induce both antigen-specific mucosal sIgA antibodies and systemic IgG antibodies, and as a result mucosal vaccines could be used in much the same way as currently available licensed parenteral vaccines [5].

*Lactobacillus plantarum* is a lactic acid bacterium (LAB) present in many ecological niches including naturally fermented food and decaying plant materials. It holds Generally Regarded As Safe (GRAS) status. *L*. *plantarum* is a normal inhabitant of the gastrointestinal (GI) tract in humans and mice, and recent genome-wide gene expression studies have identified adaptations that the bacteria use to survive in the harsh condition of the GI-tract [6–8]. It is considered a probiotic, and its high survival rate within GI-tract makes this bacterium a promising candidate for acceptability as a vehicle for *in situ* delivery of therapeutically interesting proteins [9–11]. Promising results have been achieved in inducing *L*. *plantarum* to secrete selected biomolecules, and by anchoring antigens to the cell [12,13]. Prior work has confirmed the potential of live recombinant *Lactobacillus* to deliver antigens to the immune system [4,14], suggesting the feasibility of using lactobacilli in effective oral vaccines.

FMDV invades animals primarily through mucosal surfaces, and the infection can be prevented by mucosal immune responses, suggesting that vaccines designed to elicit mucosal FMDV-specific immunity at major mucosal surfaces might interfere with viral transmission [15]. Although parenteral vaccination is usually efficient in inducing protective immune responses, the parenteral routes generally fail to activate mucosal immune responses and cannot effectively prevent the pathogens from entering the body via mucosae [15]. The creation of *L*. *plantarum* vaccines could result in halted virus transmission, and induce both mucosal immunity and systemic immunity. In this study, two recombinant *L*. *plantarum* strains—NC8 and WCFS1—were constructed to express a synthetic VP1 gene of FMDV A virus. We sought to evaluate the immunological and clinical impact of plasmids encoding FMDV-VP1 capsid protein using *L*. *plantarum* as mucosal adjuvant via oral vaccination in a guinea pig model.

## Materials and Methods

### Animal use

Female Hartley guinea pigs were obtained from Lanzhou Veterinary Research Institute (China). Female guinea pigs weighing 250 to 300g, with no maternal antibodies to FMDV, were maintained under pathogen-free conditions with free access to pathogen-free food and water. The daily food and vegetables, such as Chinese cabbage and carrots, can satisfy the nutrient demand of animals. The cage measures 100cm×60cm×30cm and is suitable for 3 guinea pigs (total 30 guinea pigs in 10 cages). They were kept in a clean, quiet room with appropriate temperature, humidity and light. The health of the guinea pigs was monitored twice daily after FMDV challenge. All guinea pig experiments were performed in a bio-safety level 3 animal facilities of State Key Laboratory of Veterinary Etiological Biology following the protocol approved by Gansu Provincial Science and Technology Department. Experiments conformed to the local (Regulations for the administration of affairs concerning experimental animals) and international (Dolan K. 2007 Second Edition of Laboratory Animal Law. Blackwell, UK) guidelines on the ethical use of animals. Guinea pigs were anesthetized by exposure to 4% isoflurane for 3 minutes prior to blood collection, saliva collection and FMDV challenge. Guinea pigs meeting criteria for euthanasia (<25% weight loss compared to weight on day of challenge), were exposed to 5% isoflurane for 60 minutes rendering animals dead or completely non-responsive followed by cervical dislocation. All surviving guinea pigs were humanely euthanized at the end of this study.

### Bacterium, plasmid, virus, and cell line

The bacterial strains and plasmids used in this study are listed in Table 1 and the strains are plasmid-free. The *E*.*coli* strains were grown in Luria-Bertani (LB) broth at 37°C in shaking flasks, and the *Lactobacillus* strains, NC8 and WCFS1, were cultured in Man-Rogosa-Sharpe (MRS) medium at 30°C or 37°C without shaking. Solid media were prepared by adding 1.5% (*w*/*v*) agar to the broth. When appropriate, antibiotics were added as follows: ampicillin– 200 μg/mL (*E*. *coli*); erythromycin– 200 μg/mL (*E*. *coli*) and 5 μg/mL (*Lactobacillus*).

**Table 1:** Bacterial strains and plasmids.

Plasmids or bacterial strains	Relevant characteristics	Source or reference
pUC57	Vector harboring synthetic VP1 gene; Ap^r^	This work
pSIP411	Expression vector; Em^r^	[16]
*E*.*coli* DH5ɑ	Cloning strains	Takara
*L*. *plantarum* NC8 (NC8 CCUG 61730)	Host strain; plasmid-free	[17]
*L*. *plantarum* WCFS1	Host strain; plasmid-free	[18]

*L*. *plantarum* WCFS1 is a single colony isolate of strain NCIMB8826. Ap^r^ and Em^r^ indicate ampicillin and erythromycin resistant gene, respectively.

A DNA fragment encoding one peptide, VP1 gene, was isolated from serotype-A FMDV (the strain was maintained and available in the Lanzhou Veterinary Research Institute (LVRI), Chinese Academy of Agricultural Sciences (CAAS)), an epidemic virulent strain originating from an outbreak in Maoming, China, during 2013, by PCR amplification according to the manufacturer’s protocols using high-fidelity Ex Taq DNA polymerse (TaKaRa Bio Inc. Japan). The following oligonucleotide primer pair was used to amplify VP1 sequence (Table 2): sense primer P1 5’- GAC ATG TCC TCC TGC ATC T -3’, antisense primer P2 5’- CAC AAA TGT ACA GGG ATG GGT -3’. The PCR products were sequenced by Shanghai Sunny Biotechnology Co., Ltd. (Shanghai, China) and chemically synthesized by GenScript Co. Ltd (Nanjing, China), which was codon optimized with *L*. *plantarum* (GenBank KR149262) and inserted into the vector pUC57 with the *Nco*I and *Xho*I restriction sites on VP1 gene’s 5’ and 3’ sides respectively. VP1 gene (647 bp) was digested out of pUC57-VP1 recombinant plasmid and inserted at corresponding sites into the plasmid pSIP411 (gift of Prof. Lars Axelsson, Nofima, Norway). Baby Hamster Syrian Kidney (BHK) cells were cultured in Dulbecco’s modified Eagle’s medium (DMEM, Gibco) supplemented with 10% (*v*/*v*) fetal bovine serum (FBS, Gibco), 100 IU/mL penicillin and 100 μg/mL at 37°C in 5% CO_2_ condition.

**Table 2:** Primers used for PCR amplification.

Oligonucleotides	Sequence (5’- 3’)	Target	Source
sense primer P1	GAC ATG TCC TCC TGC ATC T	VP1 gene	This work
antisense primer P2	CAC AAA TGT ACA GGG ATG GGT	VP1 gene	This work
sense primer P3	GCC CAT GGA AAC AAC AGC AAC AG	synthetic VP1 gene	This work
antisense primer P4	GCT CGA GTT ATA ATA ATT GTT TCG CAG G	synthetic VP1 gene	This work

(Restriction sites are underlined.)

### Transformation and electro-transformation

Chemically competent *E*. *coli* DH5ɑ (Takara) was transformed applying the protocol provided by the manufacturer. Lactobacilli were electro-transformed according to the protocol of Aukrust *et al*. but with the following modifications [17]: (1) 2% instead of 1% glycine was used in the preparation of electro-competent lactobacilli, and (2) the cells were washed three times in wash buffer (0.5mM Na-phosphate, 0.1mM MgCl_2_, pH = 7.4), and resuspended in electroporation buffer (0.1M sucrose, 3mM MgCl_2_, pH = 7.4) and stored at -80°C. A stationary phase (12 h) culture of recipient *Lactobacillus* strains, NC8 and WCFS1, were inoculated (1/100 inoculum) (*v*/*v*) into 100 mL of MRS broth (containing 2% glycine) without antibiotics at 30°C without shaking. The cells were harvested at OD_600_ 0.3 (about 3 h), put on the ice for 10 minutes, centrifuged at 2,000 r/min for 10 minutes at 4°C and washed two times with an equal culture volume of ice-cold wash buffer. Then the cells were resuspended in 1 mL ice-cold sucrose-magnesium chloride electroporation buffer (SMEB). 10 μL of recombination plasmid DNA was mixed with 200 μL of ice-cold competent cells in SMEB. The mixture was placed on ice for 5 minutes and then was transferred into a prechilled cuvette (inter-electrode distance 0.2 cm, Bio-Rad). A single electrical pulse was 2,500 V/cm, 2.5 μF and delivered via Gene Pluster^TM^ (Bio-Rad). The suspension was immediately put on ice for 5 minutes and 900 μL of recovery medium was added (MRS broth without antibiotics containing 2% glycine). The bacteria were then incubated for 3 h at 30°C without agitation and at last the recombinant strains were selected on MRS agar medium containing 5 μg/mL erythromycin.

### Protein expression and western blot analysis

For analysis of VP1 expressed by pSIP411 in NC8 and WCFS1, the recombinant strains were grown for 12 h in MRS broth containing 5 μg/mL erythromycin at 37°C without shaking. Then the overnight cultures were inoculated (1/100 inoculum) (v/v) in MRS medium and induced by adding SppIP to 100 ng/mL at OD_600_ 0.3 (about 3 h). Cells were harvested at OD_600_ 1.0 (about 7 h after induction) by centrifugation at 8,000 r/min at 4°C. The bacterial cells were washed three times with sterile phosphate-buffered saline (PBS, PH = 7.4) at 4°C and resuspended in lysis buffer (50mM Tris-HCl, 400U mutanolysin/mL, 20mg lysozyme/mL, 50 mM glucose, and Roche complete protease inhibitors) at 37°C for 1h. The bacteria were centrifuged at 8,000 r/min for 15 minutes at 4°C, and then the pellets were resuspended for analysis via 12% Sodium dodecyl sulphate-polyacrylamide gel electrophoresis (SDS-PAGE). Protein extracts were electro-transferred onto a nitrocellulose membrane for western blot analysis using swine anti-FMDV antibodies (prepared previously in our laboratory) at a dilution of 1:200 and horseradish-peroxidase (HRP)-conjugated goat anti-swine IgG antibody (Abbkine, Inc., USA) at a dilution of 1: 2,000, which was visualized with chemiluminescent substrate reagent according to the manufacturer’s instructions.

### Immunization procedures

Cells (NC8-pSIP411, NC8-pSIP411-VP1, WCFS1-pSIP411 or WCFS1-pSIP411-VP1) grown at 37°C in MRS medium containing 5 μg/mL of erythromycin, induced at OD_600_ 0.3 and harvested at OD_600_ 1.0, were washed in PBS and resuspended in milk. Five groups (n = 6) of clean guinea pigs were orally dosed with the *Lactobacillus* strains with a 1.0 mL syringe—vaccinated every 7 days with 200 μL (10^9^ CFU (colony forming unit) in 200 μL of milk for three consecutive days (i.e., the animals were vaccinated on days 1~3, 11~13 and 21~ 23), using milk as control groups (Table 3).

**Table 3:** Immunization groups.

Groups	Number	Immunizing dose (10^9^ CFU/mL)	Vaccines
**A**	6	0.2 mL	NC8 (pSIP411-VP1) + milk
**B**	6	0.2 mL	NC8 (pSIP411) + milk
**C**	6	0.2 mL	WCFS1 (pSIP411-VP1)+ milk
**D**	6	0.2 mL	WCFS1 (pSIP411) + milk
**E**	6	0.2 mL	Only milk (control group)

CFU: colony forming unit.

On days 0 (pre-immune), 10, 20 and 30, 500 μL of anticoagulated whole blood samples and 500 μL blood samples for serum tests were collected from whole blood of heart; 300 mg of fecal samples was obtained every 2 days in 300 μL sterile PBS (pH 7.4) containing 0.01 mol/L EDTA-Na_2_ and then incubated for 12 h at 4°C. Saliva samples were also collected every 2 days from the mouth of the guinea pigs using sterile rayon balls and soaked in the PBS with 1 mM protease inhibitor. Clear extracts of all samples were stored at -80°C for subsequent analysis.

### Detection of antibodies using enzyme-linked immunosorbent assay (ELISA)

Specific IgG in serum was tested using an indirect ELISA. 96-well EIA/RIA microplates (Costar; Corning, Inc.) were coated for 12 h at 4°C with FMDV-A antigen. After three times washing with PBS + Tween 20 (0.1%) (PBST), the wells were blocked for 2 h at 37°C with PBST containing 5% skim milk to prevent nonspecific binding and then were washed three times. Serum (1:10 dilution) was reacted with the coated wells for 1 h at 37°C. After the plates were washed three times, HRP-conjugated goat anti-guinea pig IgG antibody, diluted 1:5,000, was used to detect the bound antibodies, and the plates were incubated for an additional 50 minutes at 37°C. After another three washes, colour development was carried out using o-phenylenediamine (OPD) as the substrate, and optical absorbance was measured at 492 nm within 15 minutes after adding the stop buffer. For the ELISA tests in this study, cut off values were defined as the mean OD plus 3×standard deviation (SD) of samples from 30 guinea pigs used in this study before immunization.

A Capture ELISA test was used for the detection of IgA and IgM in serum as well as sIgA in saliva and feces, according to the protocol of Satya *et al*. [19]. After 96-well microplates were coated for 12 h at room temperature (RT) with rabbit antiserum against FMDV (1:1,000 dilution) and washed three times with PBST, FMDV-A antigen (1:1,000 dilution) was added to each well and incubated for 1 h at 37°C. After a further wash step, test samples were added to the wells (serum 1:10 dilution, saliva 1:4 dilution and feces 1:2 dilution) and incubated for 1 h at 37°C. After the plates were washed three times, HRP-conjugated goat anti-guinea pig IgA/IgM antibody was used to detect bound antibodies, diluted 1:1,000, and incubated for additional 50 minutes at 37°C. After three times washes, colour development was carried out using OPD as the substrate, and optical absorbance was measured at 492 nm within 15 minutes after adding the stop buffer.

### Detection of specific neutralizing antibodies against FMDV

Serum samples obtained from guinea pigs on day 30 were analyzed for neutralizing antibody titers using a neutralization assay with monolayers of BHK-21 cells [20]. 50 μL sera samples were added to each well by double ratio dilution method (from 1:2 to 1:512) with DMEM (Gibco) containing 2% FBS in a 96-well plate (before adding the sera samples, the serum was inactivated by incubating at 56°C in 30 minutes). Samples obtained from the guinea pigs immunized orally with the empty vector or milk were used as controls. 50 μL of FMDV, adjusted to 100 TCID_50_ by DMEM containing 2% FBS, was added to each cell of the plates containing serially diluted serum. The antibody and virus mixture were mixed and incubated at 37°C for 1 h. Then 100 μL of BHK cells (10^6^ cells/mL) was added to the antibody-virus mixture and incubated with 5% CO_2_ at 37°C. After 5 days incubation, CPE was measured by microscopy and each sera dilution were counted when the negative sera control and the blank control had no CPE. After the supernatant medium was discarded, 50 μL stationary liquid (10% formaldehyde) were added to each well incubated for 30 minutes and then stained with 10% formaldehyde containing 0.05% methylene blue solution for another 30 min. Read-Muench method was used to calculate the neutralizing antibody titer. The neutralizing antibody titers were calculated as the log_10_ of the reciprocal of the final serum dilution that neutralized 100 TCID_50_ of virus in 50% of the wells.

### T cell proliferation

Five guinea pigs from each immunization group (and controls) were dissected under aseptic condition, and single-lymphocyte suspensions were prepared from the spleen on day 30. Cells were labeled with carboxyfluorescein succinimidyl ester (CFSE) as described previously. CFSE-labeled cells were washed three times in PBS containing 3% FBS and then resuspended in Roswell Park Memorial Institute 1640 medium (RPMI-1640) plus 10% FBS, 15 mM HEPES and 0.05 mM ß-mercaptoethanol. 1 mL cell suspensions were added to each well of the 24-well plates at 1.0~2.0×10^6^ cells/mL. The cells were stimulated with 10 μg/mL concanavalin A (con A) (positive control), 10 μg/mL inactivated FMDV antigens (specific antigen stimulation) and culture medium (negative control), respectively, each group containing three parallel wells, incubated with 5% CO_2_ at 37°C. After 60 h of incubation, the lymphocytes were collected and concentrated by centrifugation at 1,500 r/min for 10 minutes, and then washed three times in PBS and finally resuspended in 300 μL PBS. The data for T cell proliferation was acquired with a FACSAria flow cytometer (BD, USA).

### Determination of T lymphocyte subsets

Blood samples on day 30 obtained with the anticoagulation agent EDTA were stained with R. Phycoerythrin (RPE)-conjugated mouse anti-guinea pig CD4 antibody and fluorescein isothiocyanate (FITC) conjugated mouse anti-guinea pig CD8 antibody for 30 minutes at room temperature (RT), using blood samples with RPE and FITC conjugated mouse IgG1 antibodies as negative control and blood as blank control. Subsequently, FACS lysing solution (BD FACS^TM^) was added to each sample to lyse blood cells for 15 minutes at RT. The cell pellets were resuspended by PBS after three washes, and detected by a FACSAria flow cytometer.

### Animal infections

Forty days after the first immunization, anesthetized guinea pigs were challenged subcutaneously and intradermally in the left rear leg with 0.2 mL 100ID_50_ of FMDV serotype-A. After 7 days of challenge, protection against FMDV was examined by clinical signs as follows: guinea pigs showing FMD-compatible lesions only at the original injection site were judged to be protected, and those showing any FMD clinical signs in the other three feet were judged to be unprotected [20].

### Statistical analysis

Data handling and analysis and graphic representation were performed using GraphPad Prism 6.0 software (San Diego, CA). The data were analyzed to express the mean ±SD as the error value and one-tailed *t* test was used for comparison. Differences were considered to be statistically significant when P value was less than 0.05.

## Results

### Expression of VP1 by *L*. *plantarum*

**Fig 1:**
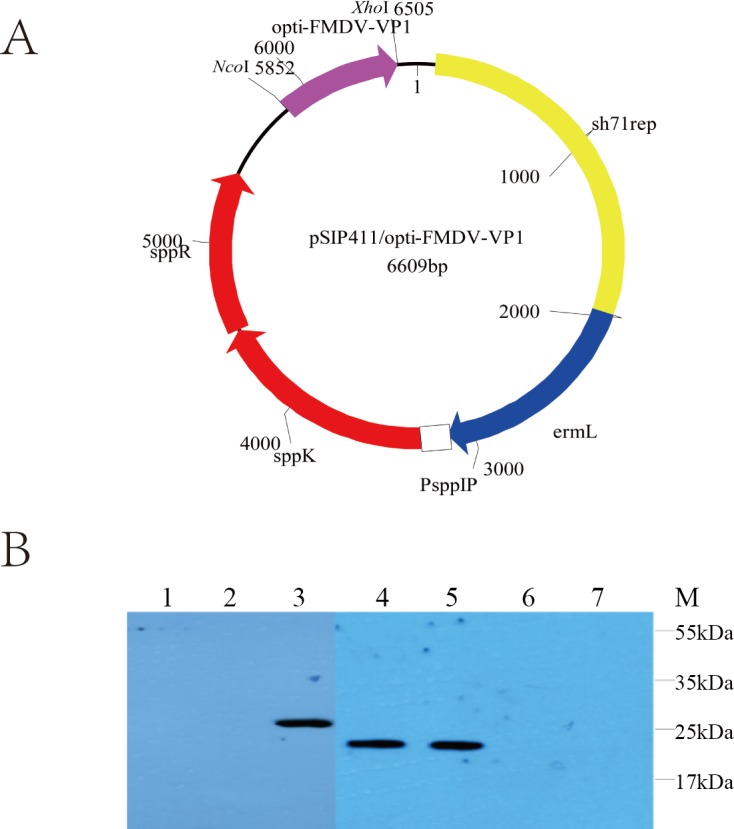
Plasmids for the expression of recombinant VP1 antigen and western blotting of expressed WCFS1-pSIP411-VP1 and NC8-pSIP411-VP1 proteins. (A) The pSIP411-VP1 plasmid was constructed as described in the article and the purple arrow stands for optimized VP1. (B) The VP1 proteins expressed in WCFS1-pSIP411-VP1 and NC8-pSIP411-VP1 were detected by western blotting. Lane 1: WCFS1-pSIP411 induced by SppIP; Lane 2: WCFS1-pSIP411-VP1 without inducer; Lane 3: WCFS1-pSIP411-VP1 induced by SppIP; Lane 4: NC8-pSIP411-VP1 induced by SppIP; Lane 5: FMDV as positive control; Lane 6: NC8-pSIP411-VP1 without inducer.; Lane 7: NC8-pSIP411 induced by SppIP. Abbreviations: sh71rep: replication origin for lactobacillus; ermL: erythromycin-resistance marker; PsppIP: inducible promoters; sppK: histidine protein kinase; sppR: response regulator.

Expression of VP1 protein by NC8 and WCFS1 cultured at OD_600_ 1.0 in basal MRS medium was induced with the inducing peptide pheromone (SppIP). The cultures were centrifuged and subjected to SDS-PAGE and immunoblotting. Fig 1 presents the expression of VP1 in NC8 and WCFS1. Immunoreactive bands of approximately 24 kDa were detected in the cell fractions of NC8 containing recombinant plasmids and the immunoreactive protein band of WCFS1 containing recombinant plasmids was about 26 kDa, whereas there were no evident bands in the negative control lanes (without plasmids and containing pSIP411).

### Antibodies responses

**Fig 2:**
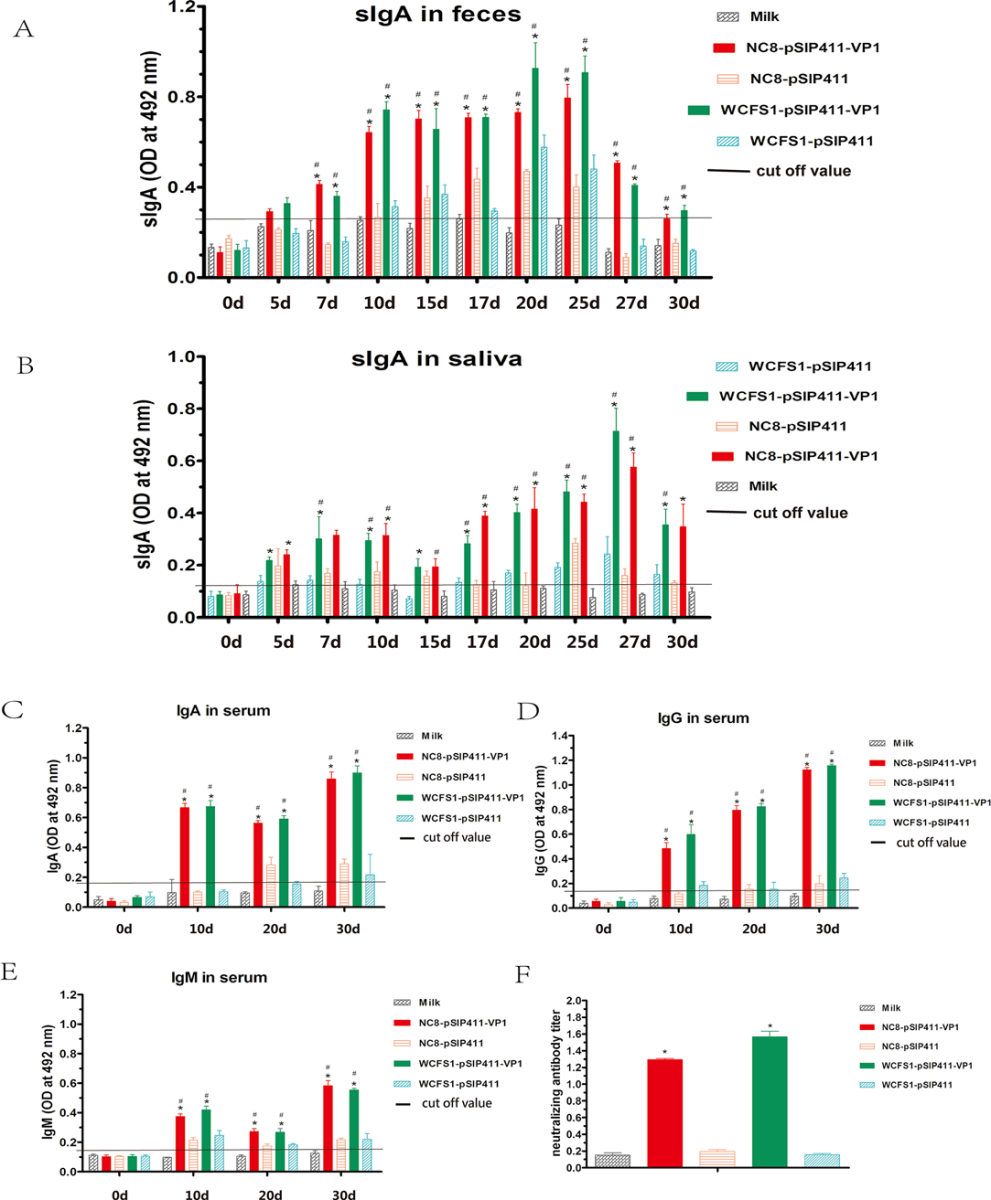
Titers of VP1-specific antibodies and neutralizing antibody. (A) sIgA antibody in feces detected by capture ELISA on days 0, 5, 7, 10, 15, 17, 20, 25, 27 and 30. (B) sIgA antibody in saliva detected by capture ELISA at 0, 5, 7, 10, 15, 17, 20, 25, 27 and 30d. (C) IgA antibody in serum detected by capture ELISA on days 0, 10, 20 and 30. (D) IgG antibody in serum detected by indirect ELISA on days 0, 10, 20 and 30. (E) IgM antibody in serum detected by capture ELISA on days 0, 10, 20 and 30. (F) Titers of FMDV-specific neutralizing antibodies. Data are expressed as mean of optical density (OD) ± SD (n = 3). “*” stands for statistically significant differences relative to the milk control and “#” represents statistically significant differences relative to the WCFS1/NC8-pSIP411 control (P<0.05). Abbreviations: Ig, immunoglobulin; SD, standard deviations.

The mucosal immunity of guinea pigs was studied by measuring the anti-FMDV-VP1 IgA response in serum. Before oral immunization on day 0, there was no substantial difference in mucosal IgA levels between the experimental (NC8-pSIP411-VP1 and WCFS1-pSIP411-VP1) and control groups (NC8-pSIP411, WCFS1-pSIP411 and milk) (Fig 2(C)) (p>0.05). Seven days following the first immunization (day 10), anti-FMDV-VP1 IgA reached high levels in experimental groups, NC8-pSIP411-VP1 and WCFS1-pSIP411-VP1 (p<0.05), while there was no obvious increase of anti-FMDV-VP1 antibody in the control groups that received pSIP411 or milk (p>0.05). Similar results for anti-FMDV-VP1 sIgA antibody were obtained for fecal and salivary samples in the immunized guinea pigs (Fig 2(A) and 2(B)). After a second immunization (day 20), the concentration of serum IgA in the experimental groups, NC8-pSIP411-VP1 and WCFS1-pSIP411-VP1, appeared lower than the original level after the first immunization (p<0.05). When compared to the mucosal IgA levels, no significant anti-FMDV-VP1 antibodies were observed in the control groups (p>0.05). The concentrations of anti-FMDV-VP1-specific IgM from immunized guinea pigs were also determined (Fig 2(E)) (p<0.05). After the third immunization, IgM antibodies rose to the highest level in NC8-pSIP411-VP1 and WCFS1-pSIP411-VP1 groups (p<0.05). Although there was no increase in the negative milk control (p>0.05), there again was a modest signal in NC8-pSIP411 and WCFS1-pSIP411 immunized animals. The FMDV-specific systemic immune responses of guinea pigs were studied by measuring the anti-FMDV-VP1 IgG response in serum. As the results show (Fig 2(D)), there was no substantial difference in IgG levels between the experimental and control groups on day 0 (p>0.05). Seven days following the first immunization (day 10), anti-FMDV-VP1 IgG obtained high levels in experimental groups, NC8-pSIP411-VP1 and WCFS1-pSIP411-VP1 (p<0.05). The concentration of IgG in the experimental groups rose further after the second immunization (day 20) and reached the highest level after the third immunization (day 30) (p<0.05). No significant elicitation of anti-FMDV-VP1 IgG antibodies was observed in the milk negative control group (p>0.05). Slight increases in IgG were again noted in the NC8-pSIP411 and WCFS1-pSIP411 groups.

Taken together, these results indicate that orally administered NC8-pSIP411-VP1 and WCFS1-pSIP411-VP1 were able to elicit both FMDV-specific systemic and mucosal antibody responses. Moreover, the mucosal immune responses occurred earlier than systemic responses but also declined more rapidly.

### Determination of T lymphocyte subsets

**Fig 3:**
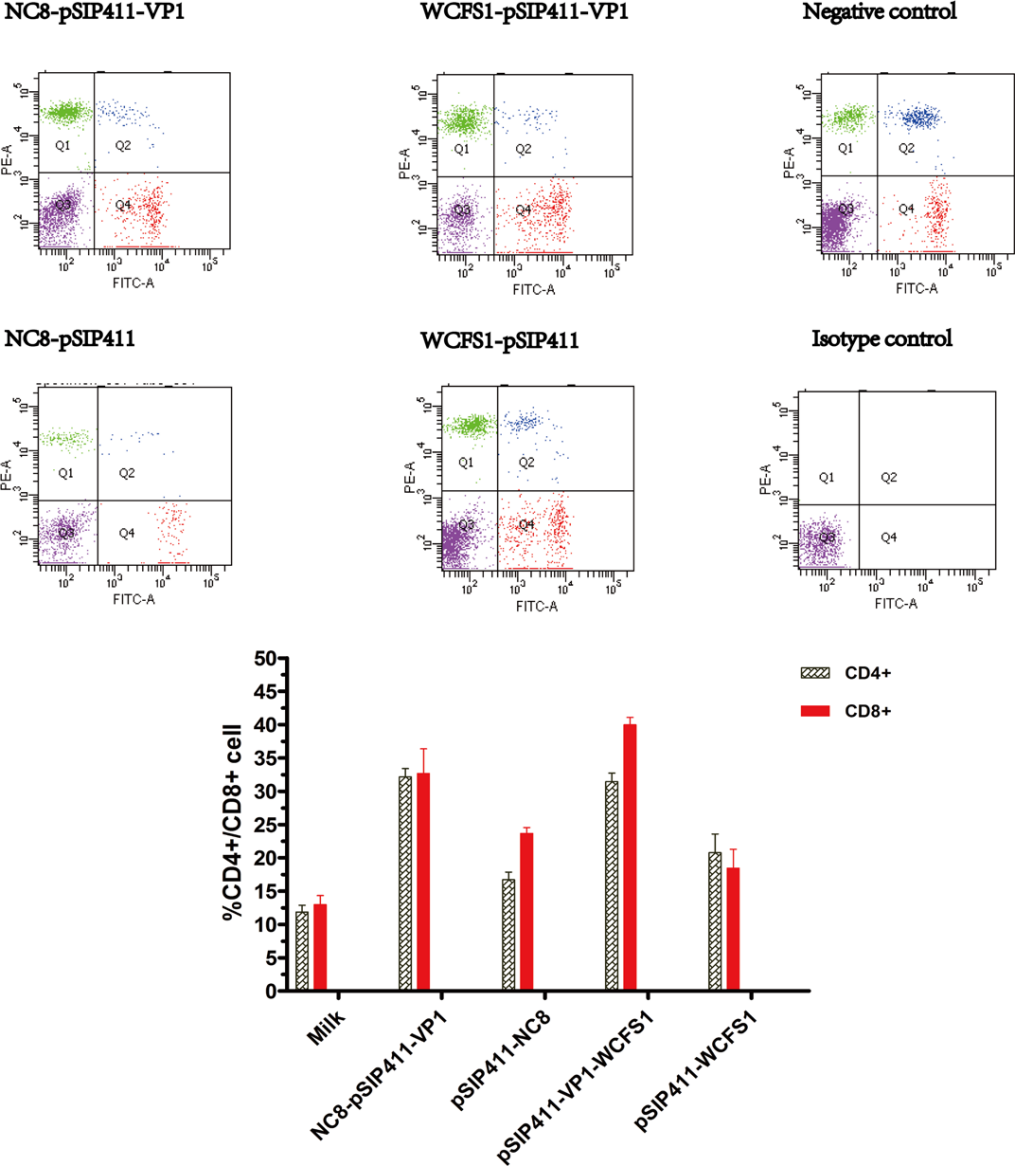
Flow cytometry for percentages of lymphocyte subpopulations between experimental and control groups. Heparinized blood from immunized guinea pigs on day 30 were double-stained with anti-CD4-RPE and anti-CD8-FITC, or incubated with the corresponding isotype controls (RPE and FITC conjugated mouse IgG1 antibodies as negative controls) for 30 minutes at room temperature. CD4^+^ and CD8^+^ T cells were analyzed as fluorescence profiles. Data are expressed as mean ± SD (n = 3). Abbreviations: Ig, immunoglobulin; FITC, Fluorescein isothiocyanate; RPE, R. Phycoerythrin; SD, standard deviations.

To investigate cellular immune responses after oral immunization with *Lactobacillus* strains incorporating the recombinant plasmid, CD4^+^ and CD8^+^ T cells were assayed by flow cytometry in blood samples obtained after the third vaccination. As demonstrated in Fig 3 the CD4^+^ T cells and CD8^+^ cells in both NC8-pSIP411-VP1 and WCFS1-pSIP411-VP1 groups rose to a higher level than the control groups.

### T cell proliferative responses

**Fig 4:**
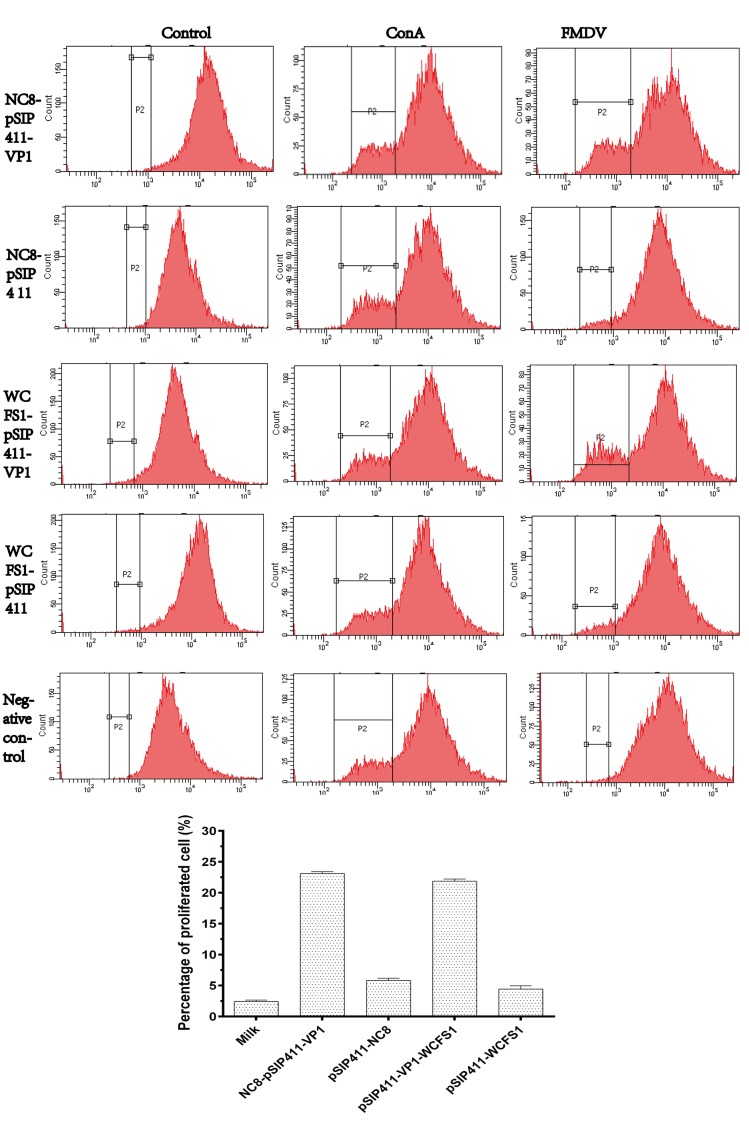
Proliferation of guinea pigs PBMCs stimulated with FMDV. PBMCs isolated from guinea pigs 30 days after the first immunization were labeled with CFSE and stimulated in vitro with inactivated FMDV, ConA (positive control), or RPMI-1640 (negative control), and plated in three replicate cultures for 60 hours. Data were acquired with FCM and analyzed with CellQuest™ software. For determination of proliferation of CFSE-stained cells, 10,000 events were captured. Data are expressed as mean percentages of proliferated cells ± SD (n = 3). Abbreviations: CFSE, carboxyfluorescein diacetate succinimidyl ester; ConA, concanavalin A; FCM, flow cytometry; FITC, fluorescein isothiocyanate; FMDV, foot-and-mouth disease virus; PBMCs, peripheral blood mononuclear cells; RPMI-1640, Roswell Park Memorial Institute 1640; SD, standard deviation.

A single-cell suspension of lymphocytes was prepared from immunized guinea pigs on day 30. After 60 h cultivation, the suspensions were stimulated by concanavalin A as a positive control—resulting in evident proliferation, and by medium as a negative control—with anticipated dormancy evident. Both NC8-pSIP411-VP1 and WCFS1-pSIP411-VP1 groups produced significantly high levels of T cell proliferation when stimulated by activated FMDV as a specific antigen (Fig 4), while no obvious proliferation occurred in the control groups. These results indicate that these *Lactobacillus* strains expressing VP1 protein could induce antigen-specific T cell responses.

### Neutralizing activity

The anti-viral neutralizing serum antibody titers of immunized guinea pigs were assessed (Fig 2(F)). Serum samples were obtained from vaccinated animals on day 30 after the first immunization. Antibody responses of guinea pigs orally immunized with NC8-pSIP411-VP1 or WCFS1-pSIP411-VP1 exhibited higher FMDV-neutralizing activity than the control groups’ responses, as shown by cytopathic effect (CPE) inhibition, indicating that NC8-pSIP411-VP1 and WCFS1-pSIP411-VP1 could induce neutralizing antibodies to FMDV.

### Protective effect of oral vaccines in guinea pigs

The guinea pigs were challenged with 100 GPID_50_ (50% guinea pigs infectious dose) of homotypic virus on day 30 after the first immunization, and the protective effects of the oral vaccines were evaluated. During ten days continuous observation post-challenge, the percentages of animals in each group exhibiting clinical signs (fever, swollen soles, and secondary vesicles) are as follows: 1/5 in NC8-pSIP411-VP1 group, 3/5 in NC8-pSIP411 group, 0/5 in WCFS1-pSIP411-VP1 group, 2/5 in WCFS1-pSIP411 and 4/5 in milk group as shown in Table 4. NC8-pSIP411-VP1 and WCFS1-pSIP411-VP1 vaccination provided at least partial protection against FMDV.

**Table 4:** Protection of guinea pigs (n = 5) against FMDV challenge.

Group^a^	Vaccines	Protection	Primary vesicles	Second vesicles
**A**	NC8 (pSIP411-VP1)	4/5	5/5	1/5
**B**	NC8 (pSIP411)	2/5	5/5	3/5
**C**	WCFS1 (pSIP411-VP1)	5/5	5/5	0/5
**D**	WCFS1 (pSIP411)	3/5	5/5	2/5
**E**	Only milk	1/5	5/5	4/5

Severe symptoms were based on daily monitoring until 7 days post challenge (one guinea pig of each group was chosen to determine the proliferation of spleen lymphocytes and the number of remaining animals in each group was 5).

^a^ Guinea pigs were challenged on day 40. Animals showing FMD-compatible lesions only at the original injection site as primary vesicles were judged to be protected, and those showing any FMD clinical signs in the other three feet as second vesicles were judged to be unprotected.

## Discussion

Although vaccines have been useful for controlling and even eradicating FMD from parts of the world since the early 1900s, the disease still infects millions of animals each year and remains the main sanitary barrier to the commerce of animals and animal products. Vaccination is the main prophylactic method of preventing FMDV infection. Many types of vaccines have been designed—almost entirely parenterally administered—including inactivated antigen vaccines, live attenuated vaccines, DNA vaccines, empty capsid vaccines and synthetic peptide vaccines [21]. Inactivated virus vaccines can elicit high levels of neutralizing antibodies and offer efficacious protection against homologous serotypes [22]. However, these vaccines have some shortcomings, including reversion to virulence, insufficient stability, and creation of viral carrier state. These vaccines cannot prevent the virus from entering the body via mucosa [15]. FMDV infection generally occurs at mucosal surfaces, which makes mucosal immunization with specific vaccine antigens an attractive option for protection against infection. Intranasal delivery of cationic PLGA nano/microparticles loaded with various FMDV DNA vaccine formulations encoding IL-6 as a molecular adjuvant enhanced protective immunity against infection by aerosolized FMDV [20]. Another study also suggested that intranasal delivery of Chi-PLGA-DNA nanoparticles with FMDV antigen resulted in high levels of mucosal, systemic and cell-mediated immunity and could reduce disease severity and virus excretion as well as delay clinical symptoms [23].

Many available approaches stimulate efficient mucosal responses, such as delivering protein or peptide antigens in association with appropriate adjuvants (e.g., liposomes and ISCOMs) [24,25], stimulating cellular or humoral immune responses in particulate form linked to latex microspheres [26] and using recombinant live vectors (e.g., attenuated virus or bacteria) [27–31]. Using lactobacilli as carriers to deliver exogenous vaccines antigens into the cytosol efficiently appears to be one of the most successful strategies [14,32]. WCFS1, the first genome annotated *Lactobacillus* strain, was found that it possessed the functionality of homologous signal peptides sequence [33]. NC8, isolated from grass silage, possessed unusual property of naturally plasmid free among *L*. *plantarum* strains [34]. These two *L*. *plantarum* can colonize the GI-tract, and are recognized as emerging candidates for the expression of recombinant proteins as well as for genetic and metabolic cell engineering [16], relevant to both medical and industrial biotechnology [35,36]. Oral administration of lactobacilli with recombinant proteins *in situ* has many advantages, such as safe, convenient, less stress among animals and reduction in adverse reaction [37,38]. Besides, lactobacilli as live vectors can persist longer in the GI-tract and some of them have intrinsic probiotic properties [39,40].

The gene used in this study, VP1 gene, has been the hot area of research in molecular biology of FMDV for years. VP1 protein exposed on the surface of viral particles is the main antigen of neutralizing antibody responses and plays an important part in forming the virus particles [41]. Over the past decades, the partial or complete VP1 coding sequence has been used in the development of engineering vaccines accommodating the large-scale production of recombinant protein, efficiently produced and expressed in various systems, such as *E*. *coli*, transgenic plants, yeasts, baculovirus and mammalian cells [19,42–46]. The sequence of VP1 gene was optimized based on the pool and codon used by *L*. *plantarum*, which reduced the GC content from 59.53% to 47.49% and without changing the gene expression, and improved the translational performance [47]. In this study, pSIP expression systems were used to express FMDV-VP1 protein. Studies are currently underway to employ lactic acid bacteria vectors to produce vaccines of many viral proteins, such as the hemagglutinin (HA) gene of H9N2 avian influenza virus, anti-rotavirus protein (Llama VHH antibody fragments), and bioactive porcine interferon-alpha [48–50]. Two *L*. *plantarum* strains were chosen in this study to carry the pSIP plasmids. Compared to the results of western blotting of these two strains, it was found that the molecular weight of target proteins expressed by WCFS1, was a little bigger than proteins expressed by NC8, on account of post-translational modification (such as phosphorylation and acetylation). Milk was used to resuspend strains in this study in order to improve taste for animals and make the oral administration safe and easy. In addition, we choose 37°C as induction temperature to imitate internal temperature of guinea pigs (the suitable temperature range of *L*. *plantarum* is 30–37°C).

In the respiratory tract, oral administration of recombinant *L*. *plantarum* can induce sIgA antibodies which are considered to be a major effector in the adaptive immune defense of the respiratory mucosa. IgA antibody emerges when antigens invade mucosa, (earlier than IgG antibody), indicating that IgA functions at the early stage of viral infection. This current study demonstrated that high levels of FMDV-specific IgA were produced in serum, saliva and feces, supporting the importance of IgA antibody responses in preventing the entry and replication of FMDV. However, the full mechanisms underlying the decrease in respiratory tract infections and other symptoms are still uncertain. The production of IgG antibody occurred later that IgA, but levels became higher and lasted longer than IgA. Taken together, the results suggested that mucosal responses induced by recombinant *L*. *plantarum* were interactive with systemic responses.

Our results are the first to report the immunization effects in guinea pigs using pSIP system with two *L*. *plantarum* host strains via oral immunization. The data we obtained clearly demonstrated that oral administration of VP1 proteins expressed by NC8 and WCFS1 could induce both mucosal responses and systemic responses and provide good protection against FMDV challenge. The mucosal administration of the vaccine strains resulted in a detectable FMDV-specific IgG and IgA responses. However, because of frequent mutations in VP1 gene region of field isolates, VP1 protein based immunization cannot produce an effective and stable immune response over a long period of time. Researches are seeking alternative genes which are carrying effective immuno-dominant epitopes and with less mutation. For example, VP2 of FMDV is believed to carry immuno-dominant epitopes, and publications revealed that VP2 protein shows important immunological characteristics in the development of FMDV vaccines [51]. Those studies are still in preliminary stage, and further studies in our laboratory will explore immune effects of other FMDV gene segments in combination immunologic adjuvants (for instance, IFN) expressed by LAB via mucosal routes in different animal models, especially in large animals.

In the past two decades, studies mucosally administered recombinant LAB (rLAB) using animal models have successfully demonstrated major health benefits, and the field has recently moved into the era of human clinical trials, which is enough to prove the potential of rLAB as therapeutic tools for their safe and efficient use. Despite good application prospect of rLAB as therapeutic delivery vehicles, there are still many questions remaining unanswered, such as the dose of therapeutic protein delivered *in situ* by the rLAB after oral administration, the more effective immunization route (the intranasal route or the oral route), the best period of life (neonate or adult)[52]. This study is in the early stages of the FMD oral vaccines and needs more tests to enhance the immune effect. Further studies will be carried on larger animals which are susceptible to FMDV, such as swine and bovine, and the studies are required to consider immune effect comparison of the intranasal immunization and the oral immunization, the coexpression of larger fragments and cytokines. Many experts anticipate that, in the next decade, more applications of rLAB as therapeutic tools will be developed and optimized because LAB delivery is now a true realistic option in both human and animal medicine [13,53,54]. In conclusion, the oral live carrier vaccine is a promising strategy for the presentation of FMDV antigen to provide an effective, inexpensive and stable alternative to current approaches of immunization for FMDV [55].
